# Enhancing Mechanical Properties: Exploring the Effect of Annealing Temperature on Wire Arc Additively Manufactured High-Strength Steel

**DOI:** 10.3390/ma16216969

**Published:** 2023-10-30

**Authors:** Yi Chen, Zhizhuang Hao, Yang Li, Chao Liu, Yongkang Liu, Zhen Luo, Sansan Ao

**Affiliations:** 1 School of Materials Science and Engineering, Tianjin University, Tianjin 300072, China; cyttbest@163.com (Y.C.); zuoxinde@tju.edu.cn (Z.H.); lz_tju@163.com (Z.L.); ao33@tju.edu.cn (S.A.); 2 College of Education, Zhejiang University, Hangzhou 310058, China; 3 Beijing Power Machinery Research Institute, Beijing 100074, China; liuyongkangvv@sina.com; 4 China Engineering Construction Welding Association, Beijing 100082, China

**Keywords:** wire and arc additive manufacturing, laminated high-strength steel, annealing, microstructure, mechanical properties

## Abstract

This study investigates the mechanical properties of exceptionally high-strength steel produced by wire and arc additive manufacturing (WAAM), using the 304 stainless steel wire and the low carbon wire (LCS). The study found that annealing treatment can enhance the steel’s mechanical properties. The microstructure in the LCS layer changed from ferrite to bainite and then to a mixture of austenite, pearlite, and bainite with increasing annealing temperature. In contrast, the SS layer retained its martensitic structure, albeit with altered lath sizes. The annealing treatment also improved the orientation of the grains in the steel. The optimal annealing temperature observed for the steel was 900 ℃, which resulted in a maximum tensile strength of 1176 MPa along the Y direction and 1255 MPa along the Z direction. Despite the superior mechanical properties, the LCS layer still exhibited failure during tensile testing due to its lower hardness. The study suggests that annealing treatment can be a useful technique for enhancing the mechanical properties of high-strength steel in WAAM applications.

## 1. Introduction

In recent years, additive manufacturing (AM) has been attracted increasing attention because of its advantages in fabricating complex-shaped metal components that cannot be produced conveniently using traditional manufacturing methods [1,2]. The most commonly used AM technology is powder bed fusion (PBF), which based on laser and electron beam power sources [3,4]. However, AM with laser and electron beams as heat sources is associated with the problem of low deposition rates, typically around 10 g min^−1^. In addition, low deposition efficiency also limits their application in fabricating large-sized components. The wire and arc additive manufacturing (WAAM) uses arc as a heat source and metal wires as the feed material [5]. This technique has a high deposition rate, up to 10 kg h^−1^, and an efficiency of 100% [6]. Therefore, WAAM is commonly used to prepare large and sophisticated parts for aerospace, automation, defense applications, and other applications [7,8].

High-strength steel is an excellent material for a wide range of engineering applications [9,10,11,12,13,14,15,16]. Traditional methods of manufacturing high-strength steel included cold rolling, heat treatment, dynamic plastic deformation treatment, and mechanical treatment [17,18,19,20,21,22,23]. However, these methods were not only complex processes with high equipment costs, limitations on the size of the part, and low production efficiency. At the same time, it is difficult to strike a balance between the strength and toughness of the prepared high-strength steel. In order to improve production efficiency and facilitate the regulation of alloy composition, many researchers have applied AM to fabricate high-strength alloys [24,25]. Fang et al. used WAAM to fabricate an 800-MPa-class high-strength low-alloy steel. It showed a good balance between strength and ductility, confirming the feasibility of WAAM for the preparation of large-sized, high-strength, and ductile block parts [26]. Jing et al. improved the anisotropy of additively manufactured 304L stainless steel by controlling the heat input of the arc and found that increasing the hot-wire current can reduce the temperature gradient in the melt pool, refine the grains, and therefore reduce the anisotropy of the material [27]. In addition to the use of WAAM technology for the manufacture of single alloys, the use of WAAM for the manufacture of metal laminates has been gradually developed. Compared with monofilament additive manufacturing, dual-filament heterogeneous material additive manufacturing can realize the gradient transition of material composition and reduce the material cost while ensuring good mechanical properties. Many scholars have conducted research on dual-filament additive manufacturing. Jin et al. used alternating feeds of high-strength, low-plasticity ZL205 and low-strength, high-plasticity 4043 wires to deposit high-strength steel. The two kinds of wire form a molten pool for full fusion to avoid defects, while the gradient of the composition of the existence of large columnar crystals [28]. Ayan et al. realized the arc additive manufacturing of functional gradient materials: two different metals (ER70S-6 and 308LSi) were used to meet the industry’s requirements for localized material properties. The strength was increased by 46% compared with a single material [29]. In our previous work, WAAM technology was used to prepare laminated materials with synergistic strength and ductility enhancement; it is twice as strong as the single material and has elongation in between. [30]. 

However, the unique thermal cycling process of additive manufacturing leads to non-uniformity in the micro-grain scale, which can have an impact on the mechanical properties, such as non-uniform strain [31]. Therefore, an overall annealing treatment is required to homogenize the grain size, eliminate residual stresses, and further improve the strength and toughness of the additively manufactured components [32,33,34]. However, few studies have investigated the effect of heat treatment on the microstructure and mechanical properties uniformity of additively manufactured laminated alloys, which are expected to have higher uniformity than additively manufactured single alloys. Therefore, this paper investigates the effect of annealing treatment on the microstructure and mechanical properties of wire and arc additively manufactured 304 stainless steel (304SS) and low carbon steel (LCS) laminated metal. 

## 2. Materials and Methods

The materials used in this research were 304 SS wires and LCS wires with diameters of 1.2 mm. The chemical compositions of these materials are listed in Table 1, and these data are provided by the material suppliers. 

**Figure 1:**
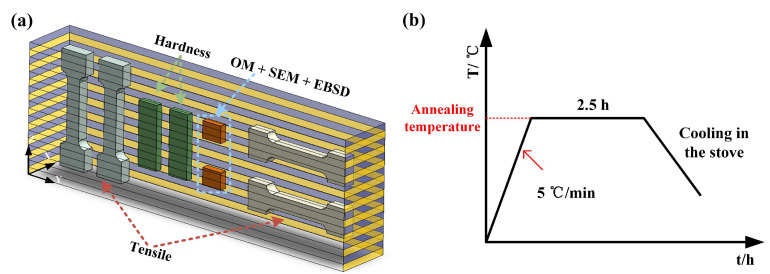
(**a**) Experimental set-up for the WAAM process and the Test sampling diagram; (**b**) Heat treatment process.

**Figure 2:**
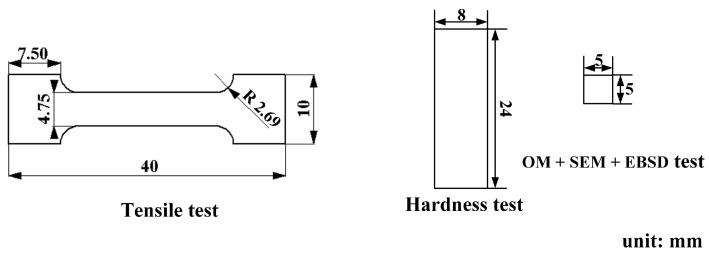
Dimensions of the testing specimens (unit: mm).

A special high-steel structure was laminated, manufactured alternately by one-layer SS and one-layer LCS. A Panasonic six-degree-of-freedom industrial robot was used in the experiments (the model and parameters of the robot are shown in Table 2), together with a Panasonic gas metal arc welder to match (the model and parameters of the welder are shown in Table 3). The welding currents for deposition LCS and 304SS layers were 100 A and 143 A, respectively, and the deposition rate was 5 mm/min. Before welding, an angle grinder and a steel brush were used to remove impurities from the surface of the substrate, followed by cleaning with acetone. Alternate layers of SS and LCS were deposited on the substrate, as shown in Figure 1a. After the deposition was completed, tensile tests and metallographic specimens were cut from the manufactured part using the wire cutting process; the locations are shown in Figure 1a. The specimens for tensile testing were cut along the Y and Z directions, where the width direction of the specimen cut along the Y direction contained at least three deposit layers. The specimen for hardness testing was a block cut along the Z direction and contained multiple deposit layers in the length direction. The specimen for microstructure observation was a smaller block but included at least two deposit layers. The dimensions of these specimens are shown in Figure 2. 

The surface of the metallographic specimens was polished with sandpaper of different grits from 200# to 2000#. A PG-1A metallographic polishing machine was used for polishing. The polished specimens were cleaned with alcohol, and the LCS layer and SS 304 layer were etched with nitric acid solution (4% nitric acid) and aqua regia (hydrochloric acid: nitric acid = 3:1), respectively. The etching time is based on the darkening time of the surface of each specimen. Mechanical tests were performed using the CSS 44,110 materials testing system. The tensile rate in the experiment was 5 mm/min.

For the two wires used in the experiment, the onset of austenite formation and the complete austenitization temperatures were about 727 °C and 950 °C, respectively. Therefore, in order to comprehensively investigate the effect of annealing temperature on the microstructure and properties of formed parts, the range of annealing temperatures selected in the experiments was from 500 °C to 1100 °C (500 °C, 600 °C, 700 °C, 800 °C, 900 °C, 1000 °C, and 1100 °C). The samples were heated to the annealing temperature at a rate of 5 °C/s and held for 2.5 h, then allowed to cool to room temperature in a furnace, as illustrated in Figure 1b. After annealing in an air atmosphere, an oxide layer will form on the surface of the specimen, which may affect the subsequent microstructure observation and performance testing. Therefore, the annealed specimens were first processed on the surface to remove the oxide layer.

## 3. Results

### 3.1. Microstructure

**Figure 3:**
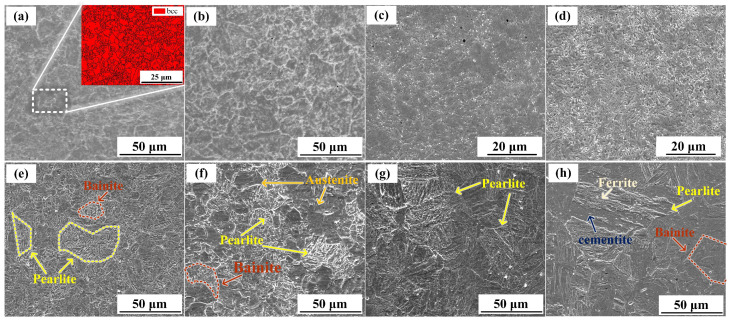
Microstructures of LCS layer: (**a**) without annealing, (**b**) annealed at 500 °C; (**c**) annealed at 600 °C; (**d**) annealed at 700 °C; (**e**) annealed at 800 °C; (**f**) annealed at 900 °C; (**g**) annealed at 1000 °C; (**h**) annealed at 1100 °C.

Figure 3a shows the microstructure of the LCS layer before annealing, consisting essentially of ferrite. The lattice structure was made up of body-center cubic (bcc) crystals. Furthermore, because of the large temperature change and unbalanced crystallization, the grain size distribution was uneven. The grain size distribution improved slightly after annealing at 500 °C and 600 °C (Figure 3b,c) [25,35]. However, the microstructure did not change due to the annealing temperatures were lower than A1 (727 °C) [36]. Thus, the important change that occurred after annealing at these temperatures was the modification in grain size. Particularly, the partial disappearance of grain boundaries (Figure 3c) is probably related to elemental diffusion from the inner grain to the boundary, such as Cr [37].

When annealing at 700 °C, the microstructure of the specimen changed significantly, and it consisted of bainite, as shown in Figure 3d. A similar phenomenon was observed by Liu et al. [38]. Figure 3e,f show the microstructures of the LCS layer after annealing at 800 °C and 900 °C, respectively. When heated to these annealing temperatures, the microstructure transformed mainly to austenite, which in turn transformed to bainite and pearlite upon cooling [39]. At 900 °C, the pearlite content was higher than that at 800 °C. Furthermore, the thickness of the pearlite layers was higher at 900 °C because of the higher diffusion rate [40]. On the other hand, the bainite content was higher at an annealing temperature of 800 °C. Untransformed austenite was also observed at an annealing temperature of 900 °C.

At annealing temperatures of 1000 °C and 1100 °C, complete austenitization was observed in the LCS layer. The austenite grain size was larger at 1100 °C compared with 1000 °C. Additionally, the higher diffusion rate at 1100 °C led to the formation of thicker layers of ferrite and cementite. In the 1100 °C annealed specimen, the pearlite lamellae spacing is larger, but the content is lower than that at 1000 °C. Higher temperatures increased both the amount of carbon dissolved in the austenite and the growth of the austenite grain. During the cooling process, these factors promoted the growth of cementite and inhibited the growth of ferrite [41]. Therefore, as shown in Figure 3h, the cementite content in the pearlite was higher than the ferrite content. 

In contrast to the complex microstructural evolution observed in the LCS layer, the microstructures observed in the SS layer at different annealing temperatures were similar, as shown in Figure 3. Figure 3a shows the microstructure of the SS layer before the annealing treatment, indicating that martensite with a BCC lattice was formed during fabrication [30]. As shown in Figure 3b–h, the annealing process changed the morphology of martensite. As the annealing temperature increased, the martensite lath thickness decreased, and this trend continued up to an annealing temperature of 900 °C. According to the Hall–Petch relationship [42], finer grains lead to higher strength. With further increases in annealing temperature, the martensite lath size increased at 1000 °C and 1100 °C (Figure 3g,h).

**Figure 4:**
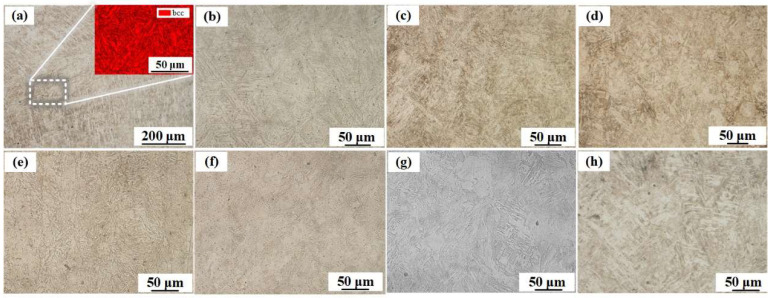
Microstructures of SS layer: (**a**) without annealing; (**b**) annealed at 500 °C; (**c**) annealed at 600 °C; (**d**) annealed at 700 °C; (**e**) annealed at 800 °C; (**f**) annealed at 900 °C; (**g**) annealed at 1000 °C; (**h**) annealed at 1100 °C.

Specimens without annealing and those annealed at 700 °C, 800 °C, and 1000 °C were further characterized using EBSD. Before annealing, both the LCS and SS layers exhibit BCC structure (Figure 3a and Figure 4a). However, the lattice structure changed after annealing. As shown in Figure 4b, traces of FCC structure with small size are observed after annealing at 800 °C. This is likely residual or untransformed austenite. There is more residual austenite in the SS layers, as shown in Figure 4d–f. This could be attributed to the presence of more austenite stabilizers in the SS layer, such as Cr and Mn [43].

**Figure 5:**
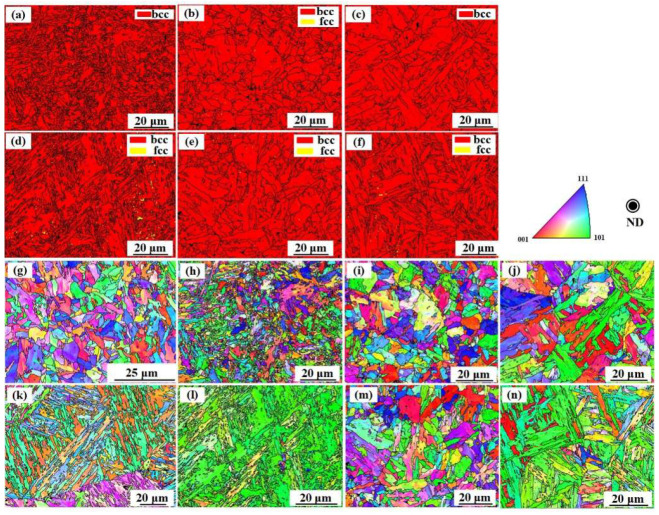
Lattice structure in the LCS layer annealed at: (**a**) 700 °C; (**b**) 800 °C; (**c**) 1000 °C; lattice structure in SS layer annealing at temperature of: (**d**) 700 °C; (**e**) 800 °C; (**f**) 1000 °C; grains orientation in layer LCS layer: (**g**) without annealing, annealed at: (**h**) 700 °C; (**i**) 800 °C; (**j**) 1000 °C, grains orientation in SS layer: (**k**) without annealing, annealed at: (**l**) 700 °C; (**m**) 800 °C; (**n**) 1000 °C.

**Figure 6:**
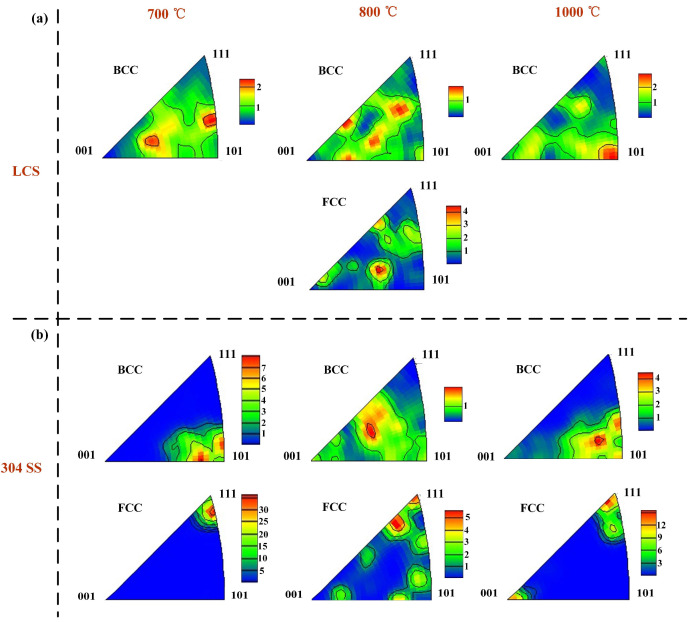
(**a**) Inverse pole figure of LCS in different annealing temperature; (**b**) Inverse pole figure of SS in different annealing temperature.

Due to the high cooling rate in the manufacturing process, the grains formed did not have a clear preferential orientation (Figure 5g,k). For the LCS layer, after annealing at temperatures of 700 °C and 800 °C, the grains exhibited a certain degree of preferential orientation (Figure 5h,i). However, after annealing at a temperature of 1000 °C, the grains were oriented mainly along the <110> direction (Figure 5j). As shown in Figure 4k–n, in the SS layer, the grains exhibit a more ordered orientation, especially the FCC structures, which are mainly oriented along the <111> direction (Figure 6b). The surface energy along the <111> direction is minimal during austenite crystallization [44]. Therefore, the grains would grow preferentially in this direction.

### 3.2. Mechanical Properties

**Figure 7:**
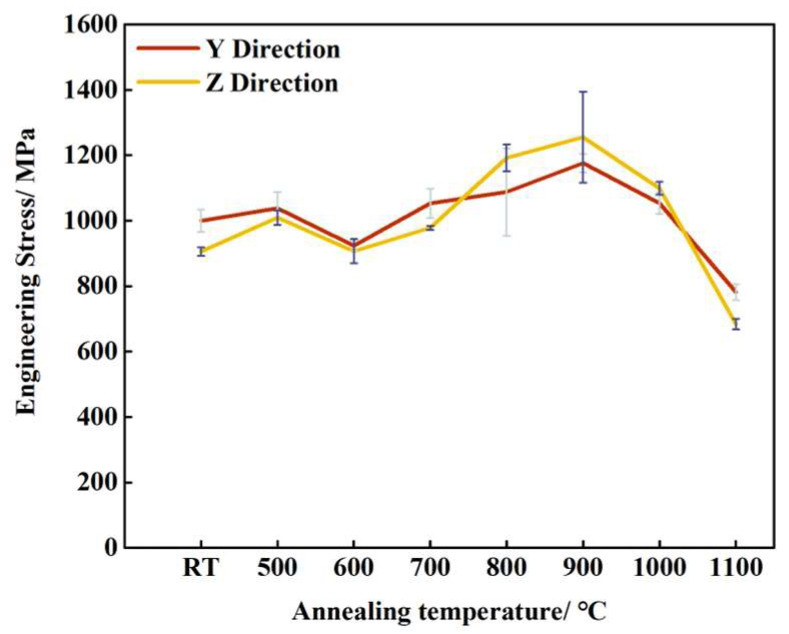
Tensile results in different annealing temperature.

The results of the tensile tests are shown in Figure 7. The change in tensile strength is mainly related to the evolution of microstructures. A reduction in grain size was observed after annealing at temperatures of 500 °C and 600 °C, thereby improving the tensile strength according to the Hall–Petch relationship. A further increase in tensile strength is observed at 700 °C due to the presence of bainite in the LCS layer [45]. At annealing temperatures of 800 °C and 900 °C, the microstructure of the specimens consists of fine, untransformed austenite, fine pearlite, and a small amount of bainite. So, the tensile strength increases compared with specimens at a lower annealing temperature. Tensile strength reached its maximum at an annealing temperature of 900 °C. The tensile strength in the Y and Z directions reached 1176 and 1255 MPa, respectively, which was 117.6% and 138.6% of the tensile strength at room temperature. With further increases in annealing temperature, the tensile strength decreased, with the lowest obtained at an annealing temperature of 1100 °C. This could be attributed to the coarsening of pearlite at high annealing temperatures. 

The mechanical properties were found to be different along the Y and Z directions at the same annealing temperature. At annealing temperatures of 500 °C, 600 °C, and 700 °C, the tensile strength in the Y direction was higher than that in the Z direction. At annealing temperatures of 800 °C, 900 °C, and 1000 °C, the tensile strength in the Z direction was higher than that in the Y direction. When the annealing temperature reached 1100 °C, the tensile strength in the Y direction was again higher than that in the Z direction. Considering the combination of tensile strength in the Y direction and the Z direction, the best mechanical properties can be achieved when the annealing temperature is 900 °C.

**Figure 8:**
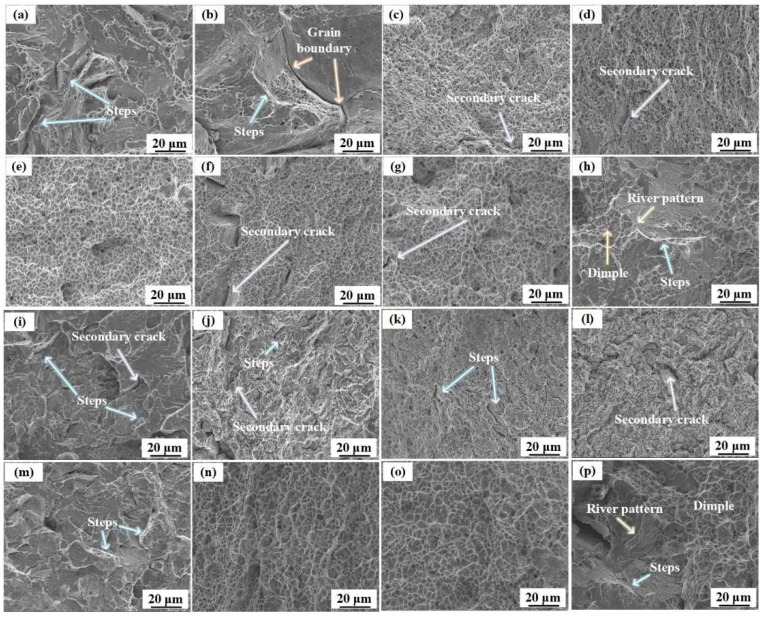
Fracture morphology in the Y direction: (**a**) without annealing; annealed at: (**b**) 500 °C; (**c**) 600 °C; (**d**) 700 °C; (**e**) 800 °C; (**f**) 900 °C; (**g**) 1000 °C; (**h**) 1100 °C; Fracture morphology in the Z direction: (**i**) without annealing; annealed at: (**j**) 500 °C; (**k**) 600 °C; (**l**) 700 °C; (**m**) 800 °C; (**n**) 900 °C; (**o**) 1000 °C; (**p**) 1100 °C.

The fracture surface morphologies of the specimens were observed using SEM. The fracture surface in the Y and Z directions is shown in Figure 8. The smallest dimples were observed at an annealing temperature of 900 °C in the Y direction, indicating that the highest tensile strength is obtained at this annealing temperature [46]. Secondary cracks were observed on the fracture surfaces of some of the specimens. This may be related to the presence of carbides [47]. At an annealing temperature of 1100 °C, the fracture surface exhibited quasi-cleave characteristics, with river patterns and dimples, as shown in Figure 8h. The fracture surfaces of the unannealed specimens and those annealed at annealing temperatures of 500 °C, 600 °C, 700 °C, and 800 °C in the Z direction exhibited the characteristics of cleavage fracture. Therefore, tensile strength in the Y direction was higher than that in the Z direction. Dimples were observed over the entire fracture surface of the samples annealed at 900 °C and 1000 °C. Furthermore, unlike in the Y direction, secondary cracks are not observed, which could be related to the higher tensile strength in the Z direction at these temperatures. At an annealing temperature of 1100 °C, quasi-cleavage fracture characteristics are observed [48], as shown in Figure 8p.

**Figure 9:**
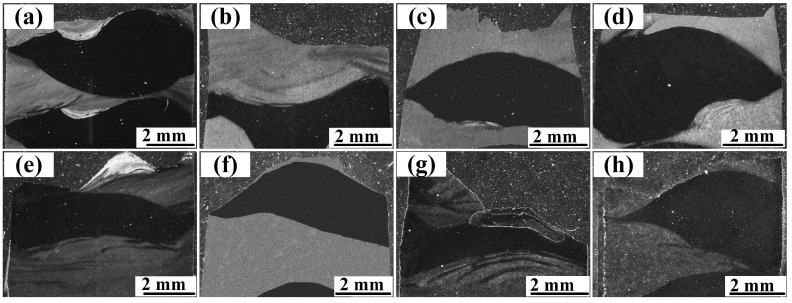
Fracture location in the samples tested along the Z direction: (**a**) without annealing, annealed at: (**b**) 500 °C; (**c**) 600 °C; (**d**) 700 °C; (**e**) 800 °C; (**f**) 900 °C; (**g**) 1000 °C; (**h**) 1100 °C.

**Figure 10:**
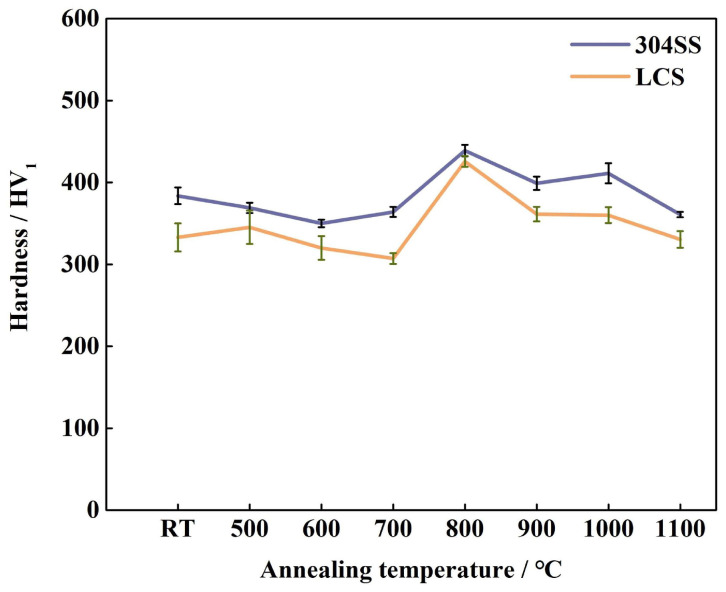
Hardness of 3304SS and LCS layers at different heat treatment temperatures.

In order to determine whether the fracture along the Z direction occurred in the SS layer or LCS layer, metallographic samples were cut from the fracture surface, as illustrated in Figure 9. It was observed that, in all specimens, the fracture occurred in the LCS layer. This is because the hardness of the LCS layer was lower than that of the SS layer, as shown in Figure 10. Therefore, plastic deformation and eventually fracture would first occur in the LCS layer during the tensile test [49].

## 4. Conclusions

This study investigated the influence of annealing temperature on the microstructure evolution and mechanical properties of laminated high-strength steel manufactured by wire arc additive manufacturing. The following conclusions can be drawn:

(1) With increasing annealing temperatures, the microstructure in the LCS layer changed from ferrite to a mixture of phases, while the microstructure in the SS layer remained martensite.
(2) As the annealing temperature increases, the grain-selective orientation in the LCS and SS304 layers gradually increases. In the LCS layer, when the annealing temperature reaches 1000 °C, the grains are predominantly oriented in the <110> direction. The FCC structure that appears in the SS304 layer after the annealing treatment is significantly oriented along the <111> direction, which is related to the minimization of the <111> surface energy during austenite recrystallization.
(3) The optimal annealing temperature for this steel was 900 °C. At this temperature, finer pearlite and martensite formed in the LCS layer and SS304 layer, respectively. The steel exhibited maximum tensile strengths of 1176 MPa along the Y direction and 1255 MPa along the Z direction, which was 117.6% and 138.6% of the tensile strength at room temperature.
(4) All Z-directional fractures in the tensile testing occurred in the LCS layer because the martensite in the SS304 layer has a higher microhardness.

## Figures and Tables

**Table 1:** Materials compositions (wt.%).

Materials	C	Mn	P	S	Si	Cr	Ni	Cu	Fe
304 SS	≤0.07	≤2	≤0.045	≤0.03	≤1	18–20	8–11	0	Bal.
LCS	0.06–0.15	1.40–1.85	≤0.025	≤0.035	0.8–1.15	0	0	≤0.5	Bal.

**Table 2:** Model and parameters of the Robot.

Type	DOF	Maximum Handling Weight (kg)	Weight (kg)	Maximum Reach Distance (mm)
YA-1VAR61CJ0	6	6	170	1437.5

**Table 3:** Model and parameters of the Welder.

Type	Rated Power (kW)	Range of Output Current (A)	Range of Output Voltage (V)
YD-350GL	13.5	40~430 (without pulse) 40~350 (with pulse)	16~35.5 (without pulse) 16~31.5 (with pulse)

## Data Availability

The data presented in this study are available on request from the corresponding author.
